# Glial cells in anorexia

**DOI:** 10.3389/fncel.2022.983577

**Published:** 2022-08-08

**Authors:** Daniel Reyes-Haro

**Affiliations:** Instituto de Neurobiología, Universidad Nacional Autónoma de México - Campus Juriquilla, Querétaro, Mexico

**Keywords:** astrocytes, microglia, eating disorders, neuroinflammation, cytokines, glutamate transporters, glutamine, prefrontal cortex

## Abstract

Anorexia is a loss of appetite or an inability to eat and is often associated with eating disorders. However, animal anorexia is physiologically regulated as a part of the life cycle; for instance, during hibernation, migration or incubation. Anorexia nervosa (AN), on the other hand, is a common eating disorder among adolescent females that experience an intense fear of gaining weight due to body image distortion that results in voluntary avoidance of food intake and, thus, severe weight loss. It has been shown that the neurobiology of feeding extends beyond the hypothalamus. The prefrontal cortex (PFC) is involved in food choice and body image perception, both relevant in AN. However, little is known about the neurobiology of AN, and the lack of effective treatments justifies the use of animal models. Glial cells, the dominant population of nerve cells in the central nervous system, are key in maintaining brain homeostasis. Accordingly, recent studies suggest that glial function may be compromised by anorexia. In this review, we summarize recent findings about anorexia and glial cells.

## Introduction

Feeding is a basic need that helps maintain energy balance and body weight. The hypothalamus, a brain region that integrates somatosensory signals and adaptive responses (neuroendocrine, autonomic and behavioral), is involved in the homeostatic regulation of feeding. The cerebral cortex is another brain region involved in feeding. Particularly, the prefrontal cortex (PFC) integrates cognitive information about food perception (odor, flavor and texture) and updates associative learning to promote (hunger) or inhibit (satiety) food-seeking behavior. The medial prefrontal cortex (mPFC) and orbitofrontal cortex (OFC) are subregions of the PFC linked with feeding, motivation for eating, foraging (Petrovich et al., 2007; Gaykema et al., 2014; Jennings et al., 2019) and anorexia (Reyes-Ortega et al., 2020). Eating disorders include anorexia nervosa (AN), a serious mental and physical illness that involves a self-distorted body image, damaging relationships with food and an obsessive desire to lose weight by refusing to eat. Adaptive anorexia, also known as animal anorexia, is part of the life cycle of vertebrates. Some examples include hibernation (bears), migration (whales) or incubation (penguins) (Mrosovsky and Sherry, 1980). Another type of adaptive anorexia is dehydration-induced anorexia (DIA), where reduced food intake occurs to ameliorate hyperosmolaemia. DIA reproduces weight loss despite food availability, and it is often used as a murine model to explore the neurobiology of anorexia. Studies using this model reported a pro-inflammatory environment associated with glial cells, which correlated with disrupted glutamate-glutamine homeostasis and neurodegeneration in the PFC of young female rats (Reyes-Ortega et al., 2022).

## Anorexia

Appetite is regulated by neural ensembles involved in homeostatic and hedonic aspects of feeding. The neurobiology of appetite is clinically relevant for eating disorders like AN. However, the term anorexia describes any loss of appetite with a concomitant reduction in food intake that occurs in the presence of readily accessible food sources. Adaptive anorexia occurs regularly at specific stages of the life cycle of animals, whereas pathological anorexia comprises AN and cachexia. Thus, neural ensembles operating in adaptive anorexia or AN may converge in a common set of synaptic circuits controlling feeding behavior.

### Adaptive anorexia

**FIGURE 1:**
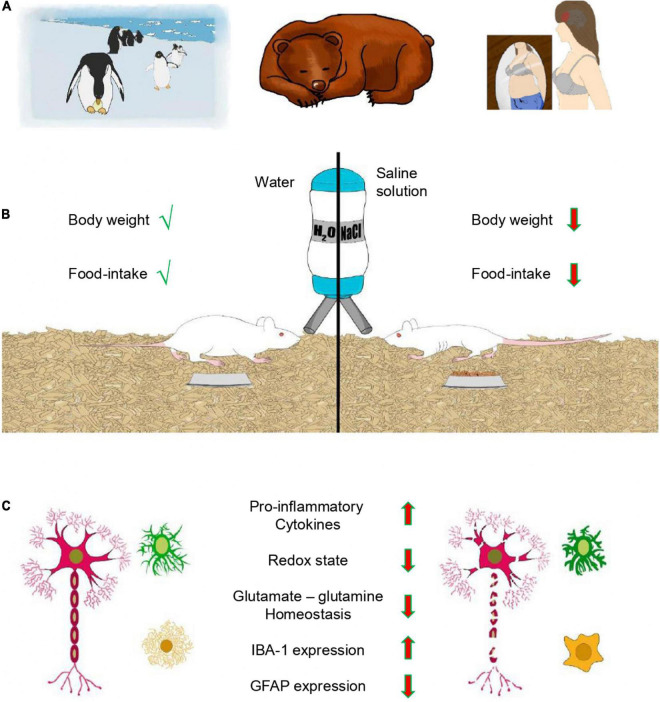
Anorexia and glial cells. **(A)** Examples of different types of anorexia. Male penguins during incubation and bears in hibernation experience adaptive anorexia (animal anorexia), which is physiologically regulated and part of the life cycle. In contrast, anorexia nervosa (AN) is pathological. **(B)** Dehydration-induced anorexia (DIA) is another example of adaptive anorexia, where animals reduce food intake in response to dehydration resulting in loss of body weight. **(C)** DIA and glial cells. Feeding provides nutrients and glial cells maintain the homeostasis of the brain. DIA induces a pro-inflammatory phenotype of glial cells with a de-ramified morphology, resulting in augmented expression of pro-inflammatory cytokines (TNFα, IL-1β, and IL-6) and the microglial marker IBA-1. These changes correlate with a decreased redox state, disrupted glutamate-glutamine homeostasis and diminished expression of the astroglial marker GFAP in the prefrontal cortex of young female rats.

Animals can adapt to predictable periods when food access is limited in nature and food intake is reduced, which often results in weight loss. However, adaptive anorexia also occurs when animals engage in other important activities that compete with feeding, even when plentiful food is available. Indeed, feeding can be disrupted by hibernation, incubation, molting, migration and defense of the harem or territory (Figure 1A). Thus, adaptive anorexia is also known as animal anorexia and it occurs regularly, at specific stages of the life cycle (Mrosovsky and Sherry, 1980). Adaptive anorexia is also evident in physiological challenges, such as cellular dehydration by water deprivation or drinking hypertonic solution (Watts, 1999).

### Anorexia nervosa

AN is a psychiatric disorder in which eating is anxiogenic and food restriction alleviates the fear of gaining weight. The onset of this mental illness occurs during adolescence and is prevalent among females (90–95%). AN is accompanied by severe weight loss, a distorted self-image and amenorrhea (Figure 1A). Moreover, it has the highest suicide rate of any psychiatric disorder, and full recovery is observed in only 21% of patients (Accurso et al., 2020).

## Murine models of anorexia

### Dehydration-induced anorexia model

Anorexia that accompanies the drinking of hypertonic saline is a critical adaptive behavioral mechanism that ameliorates hyperosmolaemia by reducing food intake (Watts and Boyle, 2010). Thus, DIA is a murine model that limits fluid loss, reproduces weight loss and reduces food intake despite its availability (Figure 1B). This model is different from other models of anorexia that impose food restriction. The effects of fasting over dehydration are distinguished with a pair-fed group under forced food restriction (FFR). The hypothalamic–pituitary–thyroid (HPT) axis adjusts to the negative energy balance induced by fasting, and the hypothalamic paraventricular nucleus promotes this adaptation by reducing the mRNA expression of thyrotropin-releasing hormone. Local T3, orexigenic and anorexigenic peptides regulate the expression of this hormone. The survival rate of the individual enhances as the serum content of thyrotropin and thyroid hormones decreases. This adaptation is observed in the FFR group, but HPT axis function fails to decrease and thyrotropin levels remain high in the DIA group of rats (Jaimes-Hoy et al., 2008; García-Luna et al., 2010, 2017). On the other hand, decreased leptin, estradiol, insulin, thyroid hormones and POMC expression, together with increased Neuropeptide Y and corticosterone serum levels, are common metabolic changes observed in FFR and DIA experimental groups (Jaimes-Hoy et al., 2008; García-Luna et al., 2010, 2017). A main feature of AN is voluntary avoidance of food, which is mimicked by DIA. Another important feature of the DIA model is that feeding and weight gain are rapidly promoted by restoring water availability (Watts, 1999; de Gortari et al., 2009; Reyes-Ortega et al., 2022). Thus, the simplicity of the DIA model allows researchers to study the cellular reorganization of ensembles in specific brain regions involved in eating disorders.

### Activity-based anorexia model

Activity-based anorexia (ABA) is a murine model that results in weight loss. This model combines food restriction to a few hours a day (1–3 h) with free access to a running wheel, resulting in a compulsive behavior that may lead to death. Food-anticipatory activity is a typical pattern observed prior feeding. The ABA model mimics some features of AN such as weight loss promoted by hyperactivity, amenorrhea, hypoleptinemia and alterations in the hypothalamus—hypophysis—adrenal axis (reviewed by Scharner and Stengel, 2021; Frintrop et al., 2022). ABA is also known to affect hormone levels; for example, cortisol, vasopressin and ghrelin concentrations are augmented while oxytocin and leptin are reduced. Insulin sensitivity is elevated because of hypoglycemia and hypoinsulinemia, and the reproductive system is disturbed by hormonal changes that disrupt the estrous cycle.

### Limitations of activity-based anorexia and dehydration-induced anorexia

The onset of AN is typically linked to psychosocial factors observed during adolescence, and relapses are common. In contrast, murine models of anorexia fail to reproduce these features, and caution must be taken when translating the results to human patients. However, ABA does reproduce hyperactivity commonly observed in AN, but it does not represent all cases and is not a diagnostic criterion. Physical activity is not prominent in DIA, but food restriction is not imposed, and this model mimics the voluntary avoidance of food intake observed in AN. Overall, both models share a negative energy balance as the main trigger of brain alterations. ABA and DIA may be complementary murine models that could help to understand the neurobiology of AN.

## Glial cells and feeding

Agouti-related peptide (AgRP) neurons of the arcuate nucleus (ARC) in the ventral floor of the mediobasal hypothalamus (MBH) are part of the circuit that drives feeding. Astrocytes modulate their activity through purinergic gliotransmission. The chemogenetic manipulation of astrocytes through hM3Dq DREADD activation showed both reduced (Yang et al., 2015) and augmented food intake (Chen et al., 2015) when the whole MBH or only the ARC was targeted, suggesting that modulation of AgRP neuron excitability by astrocytes is region-specific (Varela et al., 2021). Interestingly, Yang et al. (2015) observed anorexigenic effects after AgRP neuron inhibition through adenosine A1 receptors that were activated by adenosine derived from astrocytic release of ATP.

On the other hand, activation of hypothalamic microglia through Toll-like receptors (TLRs) elicited anorexia. These receptors participate in the innate immune system that recognizes pathogens and inflammatory signals. Interestingly, intracerebroventricular injection of the TLR2 ligand Pam3CSK4 resulted in microglia activation in the ARC. Microglial-TLR2 signaling diminished inhibitory GABAergic inputs and augmented excitatory glutamatergic inputs onto POMC neurons, resulting in increased POMC excitability (Jin et al., 2016).

### Glial cells and leptin signaling

Leptin regulates appetite and energy balance through signaling in the hypothalamus. The lack of this anorexic adipokine or the corresponding receptors results in obesity. Neurons and glial cells of the hypothalamus are known to express leptin receptors (LepRs).

Transcytosis of adipokine leptin into the brain is mediated by tanycytes through activation of LepRs that trigger calcium waves and target protein phosphorylation of the LepR-EGFR complex. In addition, EGFR-dependent activation of ERK is required for leptin transcytosis. This mechanism was confirmed after selective deletion of LepR in tanycytes because leptin entry to the brain was blocked, and increased food intake and lipogenesis were observed, as well as glucose intolerance promoted by attenuated insulin secretion from pancreatic β-cells and an imbalance of the sympathetic nervous tone. These results suggest that leptin signaling in the CNS depends on tanycyte-mediated transcytosis (Duquenne et al., 2021). LepRs expressed by dendrites in the median eminence and oligodendrocyte precursor cells (OPCs) form a metabolic ensemble that maintains leptin and weight balance. Indeed, genetic and pharmacological ablation of OPCs abolish the anorexigenic action of leptin and induce obesity in mice (Djogo et al., 2016). Dendrites at the median eminence belong to neurons of the ARC and melanocortin neurons in this hypothalamic nucleus have astrocytic coverage that regulates neuronal excitability and feeding behavior. Thus, selective ablation of LepRs in astrocytes reduced hypothalamic astrogenesis and diminished synaptic coverage due to the retraction of astrocytic processes, resulting in diet-induced obesity (Kim et al., 2014; Rottkamp et al., 2015; Wang et al., 2015). Lack of LepRs in microglia impairs phagocytosis in the paraventricular nucleus, which contributes to the decrease of POMC neurons, diminishes α-MSH projection from the ARC to the paraventricular nucleus, and increases food intake and body weight gain in mice (Gao et al., 2017). Overall, leptin signaling in glial cells is necessary for proper energy balance.

## Prefrontal cortex and anorexia

PFC activation is diminished when eating responses are inhibited in women, suggesting that this brain region may directly contribute to severe, out-of-control, maladaptive eating behaviors (Berner et al., 2022). Different fMRI studies have reported that PFC is involved in food reward and AN induces structural alterations, such as volume reduction of the mPFC (Uher et al., 2004; Mühlau et al., 2007; Brooks et al., 2011) and OFC (Lavagnino et al., 2018). The representation of taste and health attributes of food was explored in fMRI studies in the human OFC of healthy or anorexic women. The information of healthiness and tastiness was decodable from activity patterns in the OFC in both healthy and anorexic women. The representation of healthy food was stronger in anorexic women, suggesting a maladaptive over-consideration of food healthiness during deliberation about what to eat (Xue et al., 2022). The OFC is a major reward-processing hub that contains neural ensembles that respond differently to feeding or social stimuli. The activity of OFC neural ensembles, after coupling genetically encoded activity imaging with optogenetics, was monitored and manipulated at the single-cell level in real time during rewarding experiences. The results showed neural ensembles that selectively responded to either caloric rewards or social stimuli and found that optogenetic stimulation of feeding-responsive neurons was causally linked to increased feeding behavior. In contrast, optogenetic stimulation of social-responsive neurons inhibited feeding. Thus, neural ensembles within the OFC can engage to bidirectionally control feeding behavior by social stimuli (Jennings et al., 2019). On the other hand, the mPFC is known to control reward and extinction associated memories. Chemogenetic inactivation of food-reward ensembles decreased food seeking, while inactivation of the extinction ensembles promoted food-seeking behavior (Warren et al., 2016). The involvement of mPFC in hunger- and thirst-related behaviors with hydrated food and water was examined by electrophysiological recording of extracellular activity in mice. Neuronal ensembles of the mPFC distinguished hydrated food and water with similar effectiveness in hunger and thirst, and responses were strongest for the need-appropriate outcome. Thus, the mPFC was required for evaluating action-outcome relationships to inform decision-making when need states where uncertain, under variable hunger and thirst states (Eiselt et al., 2021). Considering this information, one wonders whether cellular organization of the PFC is affected by anorexia.

## Anorexia and glial cells of the prefrontal cortex

AN is linked to abnormalities of the neuroendocrine and immune systems. Leptin is a modulator of the immune response *via* microglia-induced inflammation through increased expression of tumor necrosis factor-α (TNF-α) and interleukin-1β (IL-1β) (Lafrance et al., 2010). The mRNA expression of TNF-α and IL-6 was increased in the whole blood of eleven female patients with AN (Kahl et al., 2004). Furthermore, these cytokines (TNF-α, IL-1β and IL-6) are known to inhibit feeding (Kapás and Krueger, 1992; Fantino and Wieteska, 1993; Plata-Salamán et al., 1996). Augmented expression of them correlated with increased hippocampal-microglial density in the DIA model (Ragu-Varman et al., 2019), suggesting that cognitive brain areas involved in feeding may experience neuroinflammation associated with glial cells. This option was tested in the PFC with the DIA model, and western blot studies showed an increased expression of the microglial marker IBA-1 and the pro-inflammatory cytokines TNF-α, IL-1β, and IL-6 (Figure 1C). A similar result with the FFR group indicates that increased expression of IBA-1 and pro-inflammatory cytokines is due to starvation and not dehydration (Reyes-Ortega et al., 2020). This pro-inflammatory environment resulted in a selective increase of microglial density, promoted a de-ramified morphology and augmented the de-ramified/ramified ratio in the mPFC and OFC, but not in the secondary motor cortex (M2) (Reyes-Ortega et al., 2020). Double immunofluorescence experiments with Fluoro-Jade C and the neuronal marker NeuN showed selective neurodegeneration in the mPFC (−21%) and OFC (−30%), but not in M2 (−1%). In contrast, neuronal damage was practically absent in the mPFC, OFC and M2 of the control group (Figure 1C). Thus, DIA induces a pro-inflammatory environment that involves microglia and possibly also astrocytes. Indeed, astrocytes react to starvation with a pro-inflammatory response that correlates with the expression of pro-inflammatory genes (Kogel et al., 2021). Furthermore, DIA reduced the expression of glial fibrillary acidic protein (GFAP, a classical astrocytic marker) and the number of GFAP + cells in the corpus callosum and hippocampus (Reyes-Haro et al., 2015, 2016). Similar results were reported in the corpus callosum and cortex with the ABA model (Frintrop et al., 2018, 2019), suggesting that astrocyte density in the PFC may also be reduced by DIA. Astrocytes are part of the tripartite synapse and regulate synaptic transmission by removing neurotransmitters from the synaptic cleft and releasing gliotransmitters that modulate neuronal activity (Araque et al., 1999). Neurons receive metabolic support from astrocytes (Rouach et al., 2008), and the glutamate-glutamine cycle between them is a major metabolic pathway that requires ∼80% of the brain’s total glucose consumption (Magistretti and Pellerin, 1999; Shen et al., 1999). Glutamate, the main excitatory neurotransmitter in the brain is removed from the synaptic cleft by high-affinity transporters expressed mainly in astrocytes. This mechanism ends synaptic transmission and prevents excitotoxicity (Reyes-Haro and Salceda-Sacanelles, 2000; Danbolt, 2001). Astrocytic glutamate transporters are sodium dependent, and their function is coupled to augmented metabolism (Magistretti and Pellerin, 1999). Thus, glutamate is removed from the synaptic cleft by astrocytes and rapidly amidated to glutamine by ATP-dependent glutamine synthetase. Glutamine is released through system N amino acid transport and incorporated by neurons through system A sodium-coupled amino acid transport, and converted to glutamate by mitochondrial glutaminase (Bröer and Brookes, 2001). In addition, glutamate can be oxidized for energy *via* the tricarboxylic acid (TCA) cycle and pyruvate recycling pathway (McKenna, 2013). Anorexia depletes energy reservoirs, and glutamate-glutamine catabolism may help to contend with the metabolic requirements of the brain. In support of this hypothesis, DIA reduced the redox state, as well as the endogenous levels of glutamate and glutamine in the PFC. Furthermore, DIA also diminished the expression of GFAP, glutamate transporters (GLT-1 and GLAST) and glutamine synthetase; all of them are astrocytic markers associated with glutamate-glutamine homeostasis (Reyes-Ortega et al., 2022) (Figure 1C). Accordingly, astrocytic density was selectively reduced and a de-ramified morphology was promoted by DIA in the mPFC and OFC but not in M2 (Reyes-Ortega et al., 2022) (Figure 1C). The model of DIA, a type of adaptive anorexia, shows that glial cells are central to understanding the cellular mechanisms linked to starvation in cognitive brain regions associated with feeding, such as the OFC and mPFC.

## Discussion

The PFC belongs to the mesocorticolimbic reward circuit and OFC participates in the decision to eat (Gutierréz et al., 2010), while the mPFC regulates food intake (Land et al., 2014; Prado et al., 2016). Accordingly, the DIA model induces neurodegeneration associated with pro-inflammatory microglia in the mPFC and OFC (Reyes-Ortega et al., 2020). This in agreement with *postmortem* studies in girls diagnosed with anorexia showing signs of degeneration in cortical neurons (Neumärker et al., 1997). The pro-inflammatory environment induced by DIA correlated with reduced astrocyte density (Reyes-Ortega et al., 2020, 2022). Similarly, cortical astrocyte density was diminished by ABA (Frintrop et al., 2018; Hurley et al., 2022), suggesting that astrocyte loss may be relevant for the neurobiology of anorexia. In support of this hypothesis, recent studies showed that DIA promoted de-ramification of astrocytes and reduced the expression of glutamate transporters (GLAST and GLT-1). Moreover, suppression of hyperactivity correlated with augmented expression of GLT-1, minimizing the severity of ABA (Bilash et al., 2021). Thus, reverting glial cell-mediated neuroinflammation and boosting the expression of glutamate transporters may ameliorate DIA/ABA vulnerability. Overall, we propose that glial cells should be considered as a potential therapeutic target for AN, based on the experimental evidence provided by murine models of anorexia.

## Author contributions

DR-H wrote the manuscript, designed the content of the manuscript, and approved the submitted version.
